# Chromosomal-level assembly of *Magnusiomyces clavatus*: novel genetic insights on an emerging fungal pathogen

**DOI:** 10.1093/g3journal/jkaf201

**Published:** 2025-09-03

**Authors:** Emil Tonon, Riccardo Cecchetto, Maya Carrera, Annarita Sorrentino, Guido Zeni, Giona Turri, Marco Mantoan, Asia Palmisano, Anna Lagni, Virginia Lotti, Erica Diani, Davide Gibellini

**Affiliations:** Department of Diagnostic and Public Health, Division of Microbiology, University of Verona, Verona 37134, Italy; UOC Microbiology, AOUI Verona, Verona 37134, Italy; Department of Diagnostic and Public Health, Division of Microbiology, University of Verona, Verona 37134, Italy; UOC Microbiology, AOUI Verona, Verona 37134, Italy; UOC Microbiology, AOUI Verona, Verona 37134, Italy; Istituto Zooprofilattico Sperimentale della Lombardia e dell’Emilia Romagna, Virology Unit, Brescia 25124, Italy; UOC Microbiology, AOUI Verona, Verona 37134, Italy; UOC Microbiology, AOUI Verona, Verona 37134, Italy; UOC Microbiology, AOUI Verona, Verona 37134, Italy; Department of Diagnostic and Public Health, Division of Hygiene and Preventive, Environmental and Occupational Medicine, University of Verona, Verona 37134, Italy; Department of Diagnostic and Public Health, Division of Microbiology, University of Verona, Verona 37134, Italy; Department of Diagnostic and Public Health, Division of Microbiology, University of Verona, Verona 37134, Italy; Department of Diagnostic and Public Health, Division of Microbiology, University of Verona, Verona 37134, Italy; Department of Diagnostic and Public Health, Division of Microbiology, University of Verona, Verona 37134, Italy; UOC Microbiology, AOUI Verona, Verona 37134, Italy; Department of Diagnostic and Public Health, Division of Microbiology, University of Verona, Verona 37134, Italy; UOC Microbiology, AOUI Verona, Verona 37134, Italy

**Keywords:** whole-genome sequencing, *Magnusiomyces clavatus*, geotrichosis, fungal genome, emerging pathogen, nosocomial infection, antifungal resistance, phylogenetic clustering, fungi

## Abstract

*Magnusiomyces clavatus* is an emerging opportunistic fungal pathogen associated with severe systemic infections in immunocompromised patients, mostly among those suffering from hematological malignancies. Despite the increasing clinical significance, genomic data for *M. clavatus* remain limited. In this study, we report the first chromosomal-level genome assembly of *M. clavatus* using hybrid sequencing with Illumina and Oxford Nanopore Technologies. Three clinical isolates obtained from ICU patients in Verona (Italy) were sequenced and analyzed. The *M. clavatus* genome was resolved into 4 nuclear chromosomes and 1 circular mitochondrial genome, with a total length of 17.6 Mb and with 4,065 predicted protein-coding genes. Comparative analyses revealed structural differences from its closely related species *M. capitatus*. Phylogenetic analysis of 40 strains assembled on the resolved genome identified a novel clade (D), distinct from the previously described clades A, B, and C. All isolates exhibited intrinsic resistance to echinocandins and fluconazole. Genetic analysis identified conserved mutations in the *FKS1* hotspot region encoding 1,3-β-glucan synthase, mirroring resistance-associated substitutions in *M. capitatus*. Additionally, a putative *cyp51* homolog was identified as a likely contributor to azole resistance, suggesting conserved resistance mechanisms across *Magnusiomyces* species. This study discloses a new chromosomal-level assembly for *M. clavatus*, providing a significant genomic framework. This resource could enhance the accuracy of diagnostic methods, enabling comparative genomics with closely related fungi and facilitating a deeper investigation into the mechanisms of antifungal resistance and pathogenicity of this rare but increasingly reported pathogen.

## Introduction

*Magnusiomyces clavatus*, formerly known as *Geotrichum clavatum* or as *Saprochaete clavata* (De Hoog et al. 1986; Verkley 1999; De Hoog and Smith 2004), is a pathogenic arthroconidial yeast-like fungus closely related to *Magnusiomyces capitatus* (previously known as *Saprochaete capitata*, *Geotrichum capitatum*, *Blastoschizomyces capitatus*, *Trichosporon capitatum*, or *Dipodascus capitatus*) (Zhu et al. 2024). These 2 human pathogens are often misidentified from each other because of their similar phenotypes. The most reliable methods for discriminating between the 2 species are ITS sequencing and, more recently, matrix-assisted laser desorption/ionization-time of flight mass spectrometry (MALDI-TOF MS) (Desnos-Ollivier et al. 2014; Noster et al. 2022).

Both fungi are considered emerging threats for immunocompromised patients, especially those with hematological malignancies and neutropenia (Ulu-Kilic et al. 2015; Buchta et al. 2019). *M. clavatus* and *M. capitatus* are intrinsically resistant to echinocandins, which are recommended in cases of *Candida* fungemia or febrile neutropenia of unknown origin in hematology wards (Freifeld et al. 2011). Systemic infections associated with these 2 pathogens show a high mortality rate, from 40% up to 80% (Durán Graeff et al. 2017; El Zein et al. 2020). Risk factors for geotrichosis, an infection associated with *Geotrichum* species (including *M. clavatus* and *M. capitatus*), are AIDS, uncontrolled diabetes, and malignancies (Chen et al. 2021). The most common conditions in immunocompromised patients are bloodstream and pulmonary infections (Durán Graeff et al. 2017), even though localized forms such as onychomycosis can also occur (D’Antonio et al. 1999).

The epidemiology of both pathogens appears to be influenced by climatic factors, with the majority of cases occurring in the Mediterranean region (Birrenbach et al. 2012). Although invasive infections caused by *M. capitatus* are present in the literature from 1977 (Winston 1977), both in humans and animals (AalbæK et al. 1994), the number of cases has increased over the past 2 decades. More recently, cases of *M. capitatus* have been reported in Central Europe as a possible consequence of global warming (Garcia-Solache and Casadevall 2010; Birrenbach et al. 2012).

*M. clavatus* and *M. capitatus* caused multiple outbreaks, particularly in Italy and France, with the largest cluster in 2013 involving *M. clavatus* and accounting for 39 cases (Camus et al. 2014; Picard et al. 2014; Vaux et al. 2014; Lo Cascio et al. 2020). The natural reservoir remains elusive, as *M. capitatus* has been found on hospital sanitation equipment as well as in the environment, such as in soil or decaying wood (Randhawa et al. 2001). In a French *M. capitatus* outbreak that occurred in 2002, the source of contagion was milk vacuum flasks used for breakfast milk distribution (Gurgui et al. 2011). However, *M. capitatus* has also been reported to be present on the skin of some immunocompetent subjects (D’Antonio et al. 1999), and data from Vaux et al. demonstrate the colonization of the intestine from *M. clavatus* in at least 1 case of an asymptomatic patient (Vaux et al. 2014). In addition, during an outbreak at a cancer center in Marseille, the source of *M. clavatus* nosocomial infection was traced to a dishwasher with a deficient heating system (Menu et al. 2020).

While extensive genomic data are available for *M. capitatus* (Opulente et al. 2023), genetic information regarding *M. clavatus* is still incomplete, highlighting the need for further sequencing studies to increase the understanding of pathogenic mechanisms and improve diagnostic and therapeutic strategies. In this study, whole-genome sequencing (WGS) was performed on 3 clinical isolates, and a chromosomal-level genome of *M. clavatus* was provided.

## Materials and methods

### Sample isolation and susceptibility test

**Fig. 1.**
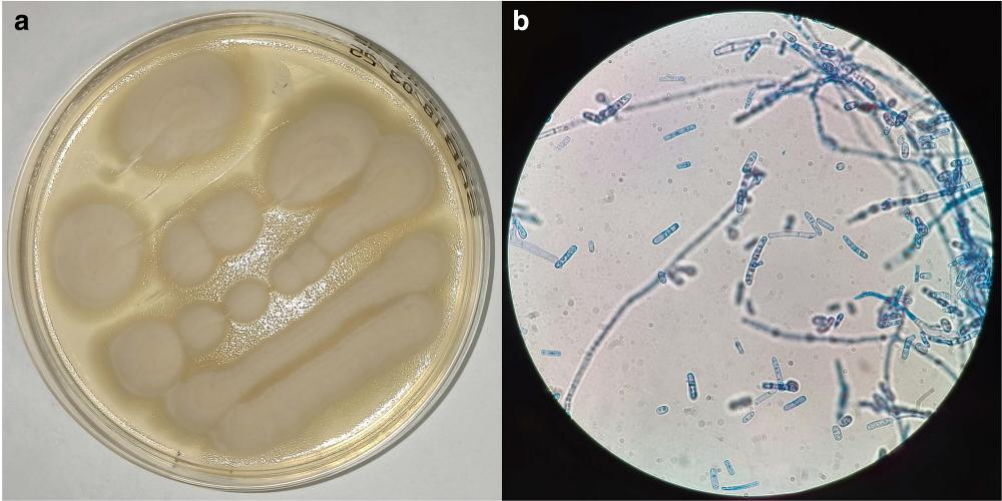
*M. clavatus* strain cultured for Illumina and Nanopore sequencing. a) *M. clavatus* growing on Sabouraud agar. b) *M. clavatus* lactophenol cotton blue stain, 100× magnification.

Specimens were isolated from bronchial aspirate (BASP) and blood samples of 3 ICU patients (strains VRMC001, VRMC002, and VRMC003) of AOUI Verona. Samples were collected between July and October 2024 and identified as *M. clavatus* with MALDI-TOF MS. Fungal strains were cultured on Sabouraud agar at 30 °C. Fungal morphology is shown in Fig. 1.

Antifungal susceptibility test was performed using the Sensititre YeastOne YO10 kit (Thermo Fisher Scientific, Waltham, Massachusetts, United States). In detail, anidulafungin, micafungin, caspofungin, flucytosine, posaconazole, voriconazole, fluconazole, and amphotericin were tested. The raw MIC data are shown in Supplementary Fig. 1 (panels a, b, and c).

### DNA extraction

DNA was extracted with Quick-DNA Fungal/Bacterial Miniprep Kit (Zymo Research, Irvine, California, United States) following the manufacturer’s instructions. Briefly, 100 mg of wet-weight fungal cells were resuspended in 200 µl of water and transferred to a ZR Bashing Bead Lysis tube with 750 µl of BashingBead Lysis Buffer. Tubes were left on a Hula mixer for 20 min; samples were subsequently centrifuged at 10,000 × *g* for 1 min. The supernatant was then transferred to a Zymo-Spin III-F Filter and centrifuged at 8,000 × *g* for another minute. Subsequently, 1,200 µl of genomic lysis buffer was added and centrifuged at 10,000 × *g* for 1 min in 2 separate steps in a Zymo-Spin IICR Column. The flow-through was discarded, and 200 µl of DNA prewash buffer was added to the column and centrifuged at 10,000 × *g* for 1 min. At this point, 500 µl of gDNA wash buffer was added to the same column and centrifuged again under the same conditions as in the previous step. Finally, the Zymo-Spin IICR Column was transferred to a clean 1.5 ml tube, adding 35 µl of DNA elution buffer, and centrifuged at 10,000 × *g* for 30 s. Total DNA concentrations were quantified via Qubit 4 Fluorometer (Thermo Fisher Scientific, Waltham, Massachusetts, United States) and were then used for Illumina and Nanopore sequencing.

### Illumina sequencing and Nanopore sequencing

Library preparation was performed with the Illumina DNA Prep Assay kit (Illumina, San Diego, California, United States) with a random primer pool. Samples were sequenced with the Illumina MiSeq instrument in paired-end mode (2 × 150 bp) with V3 chemistry. The Nanopore library was prepared using the SQK-LSK114 kit (Oxford Nanopore Technologies, Oxford, United Kingdom) in combination with the NEBNext Companion Module (New England Biolabs, Ipswich, Massachusetts, United States) and subsequently quality-checked and sequenced on a MinION Mk1B device (Oxford Nanopore Technologies, Oxford, United Kingdom).

### Bioinformatic analysis

Short-read sequencing using Illumina technology was performed on the 3 samples of *M. clavatus*. Given the short Illumina reads, the complete genome of this pathogen cannot be resolved, as demonstrated by previous attempts by Vaux et al. (2014). Hence, a long-read sequencing with Nanopore technology on strain VRMC001 was performed as well.

The Illumina quality control of reads was assured with FastQC v0.12.1 (Andrews 2010), whereas Fastplong v0.24.0 (Chen et al. 2018) was employed for Nanopore long reads. Illumina reads were trimmed using Trimmomatic v0.39 (Bolger et al. 2014) as in the study of Kraft et al. (2023), changing MINLEN 40 for single-end sequences and MINLEN 70 for paired-end sequences.

Nanopore reads were trimmed with Porechop v0.2.3 (Bonenfant et al. 2023) tool and then corrected with Illumina trimmed reads using LoRDEC v0.9 tool (Salmela and Rivals 2014).

Before the assembly, raw genome size was estimated to be around 18.9 megabases (Mb) with a *k*-mer counting heterogeneity of 13% (*k*-mer length 21) with Meryl v1.3 (Miller et al. 2008) and visualized with GenomeScope 2.0 (Ranallo-Benavidez et al. 2020). GenomeScope profile for Illumina reads is reported in Supplementary Fig. 2. Strains were considered haploid for all sequencing analyses.

De novo assembly was performed with Flye v2.9.5 assembler (Kolmogorov et al. 2019) on the Galaxy platform (Abueg et al. 2024). Given the evolutionary relatedness to *M. capitatus* (Zhu et al. 2024), the sequence of the NRRL Y-17686 strain, published by Brejová et al. (2019), was used as a scaffold for RagTag v2.1.0 tool (Alonge et al. 2022), and a further improvement of the original assembly was obtained, reaching a full chromosomal-level genome. To obtain a better resolved assembly, especially for ribosomal repeats, an iteration of Pilon v1.20.1 (Walker et al. 2014) was then performed.

Sequence-based alignment was performed using Samtools v1.17 (Danecek et al. 2021) and minimap2 v2.26 (Li 2018).

A manual check searching for possible sources of contaminants using the Bandage v9.0 tool (Wick et al. 2015) and Integrative Genomics Viewer (IGV) (Robinson et al. 2011) was performed. Any contig that did not align with the CNRMA12.647 reference was searched in the BLAST database. It was excluded if it aligned with genomes of other species with a higher score than *M. clavatus*. A final quality control was performed through Merqury v1.3 (Rhie et al. 2020), Quast v5.0.2 (Gurevich et al. 2013), and Benchmarking Universal Single-Copy Orthologs (BUSCO) v5.8.0 (Simão et al. 2015). Merqury *k*-mer count graph for Illumina reads and the final assembly is shown in Supplementary Fig. 3.

Complete sequences were annotated using Funannotate v1.8.17 (Palmenr and Stajich 2020), InterProScan v5.73 (Jones et al. 2014), and eggNOG v5.0 (Huerta-Cepas et al. 2019). Clusters of Orthologous Genes (COG) (Tatusov 2000) annotation results obtained from eggNOG were selected for COG classification statistics and mapping.

Whole-genome SNP-based phylogenetic analysis was performed on a total of 946 filtered SNPs from 40 sequences, assembled in aligned pseudo-sequences using a customized pipeline (https://github.com/et-univr/VCFtoAlignfasta2/tree/main). SNPs were obtained from regions with a minimum of 20 reads mapped in each orientation, with mapping quality of at least 50, and supported by at least 80% of the reads. Phylogenetic reconstruction was carried out using the maximum likelihood method in IQ-TREE (Wong et al. 2025). Branch support was assessed with 100 bootstrap replicates.

## Results

**Fig. 2.**
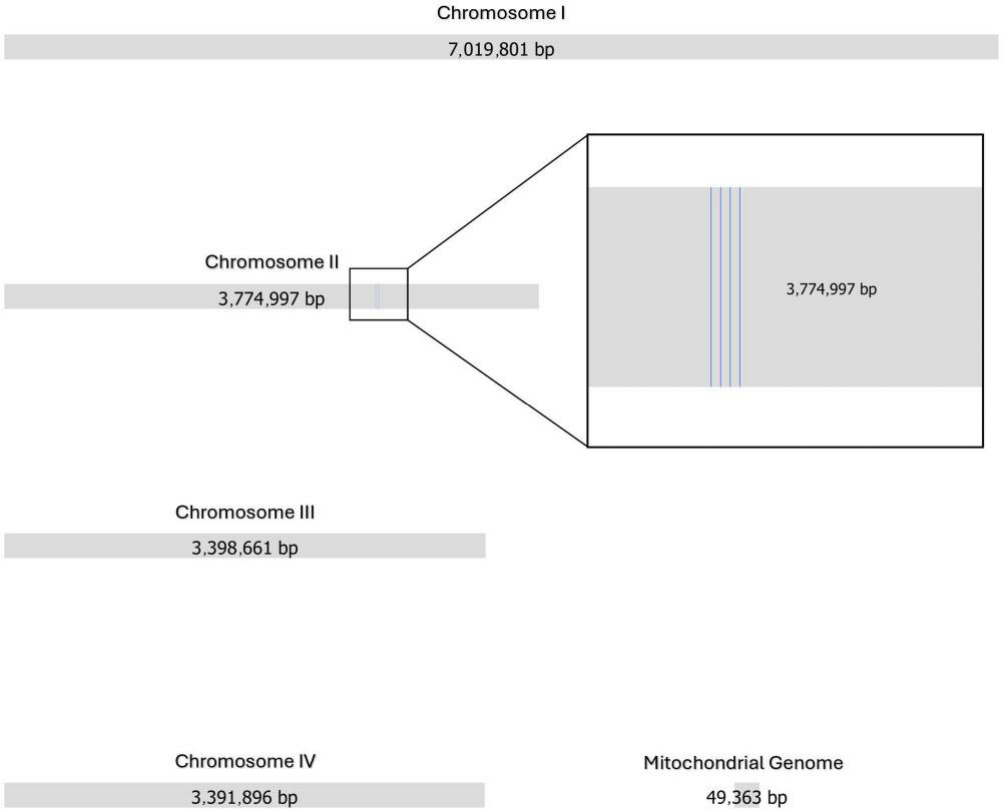
Bandage representation of the VRMC001 assembled genome with the corresponding length for each contig. In chromosome 2, the regions hosting the ribosomal genes are highlighted. Only 4 copies are included, but the real number could be estimated to be at least 100 copies (Miyazaki and Kobayashi 2011), based on the ratio between the coverage of this region and the average coverage.

To determine the whole-genome sequence of *M. clavatus* VRMC001, de novo assembly was performed using Illumina and Nanopore reads. The bioinformatic analysis returned a complete genome, divided into 5 contigs and corresponding to 4 entire chromosomes and 1 copy of the mitochondrial genome. The Bandage graphical assembly is presented in Fig. 2. Reference-based assembly of VRMC002 and VRMC003 was performed using Illumina reads, with the VRMC001 assembly obtained in the previous step as a reference.

Chromosome 1 has a total length of approximately 7 Mb and a GC content of 34.01%; chromosome 2 is nearly 3.8 Mb, but its size is estimated to be at least 3 Mb longer, as this chromosome contains the 28S region which, according to the sequencing depth, is repeated at least 100 times. A previous study indicated that these repetitions could reach up to 200 copies in *S. cerevisiae* (James et al. 2009). Chromosome 2 GC content is 34.21%. Chromosome 3 and chromosome 4 are approximately 3.4 Mb long with a GC content of 33.90% and 34.16%, respectively. The shortest contig, approximately 50 kb in length, is circular and shares multiple sequence similarities with the mitochondrial genome of *M. capitatus* (NCBI Reference Sequence: NC_024095.1) (Supplementary Fig. 4). Its GC content is 19.81%.

For each chromosome, BLAST was performed against the *M. capitatus* reference genome (NCBI entry GCA_030571335.1) to assess homology. Results are reported in Table 1. A BLAST search was performed using the 28S ribosomal RNA gene sequences, yielding 100% identity with several *M. clavatus* strains, thereby confirming the initial identification obtained by MALDI-TOF MS. When assessing completeness using BUSCO score (the percentage of the 2,137 BUSCO genes identified in the final assembly), the genome achieves 86.8% completeness (1,855 complete genes out of 2,137), compared to 86.6% for *M. capitatus* reference genome NRRL Y-17686 (1,851 out of 2,137) and 86.7% for *M. clavatus* reference genome CNRMA 12.647 (1,852 out of 2,137). In comparison, the reference genome of *Candida albicans* SC5314—the genome of one of the most studied pathogenic yeasts—demonstrates a BUSCO completeness score of 98,4% (2,103 out of 2,137). Quast quality statistics for the 3 sequences are summarized in Table 2.

**Table 1.** Chromosome-by-chromosome BLAST results of the *M. clavatus* VRMC001 genome against the *M. capitatus* genome (NCBI accession GCA_030571335.1).

VRMC001 against GCA_030571335.1	Coverage (%)	Identity (%)
Chromosome 1	31	84.75
Chromosome 2	47	83.86
Chromosome 3	32	86.31
Chromosome 4	39	82.02
Mitochondrial genome	42	92.12

**Table 2.** Summary of QUAST assembly statistics of VRMC001 compared to the *M. capitatus* reference NRRL Y-17686 and the *M. clavatus* reference sequence CNRMA 12.647.

	*M. clavatus* VRMC001	*M. capitatus* NRRL Y-17686 (Brejová et al. 2019)	*M. clavatus* CNRMA 12.647 (Vaux et al. 2014)
# contigs	5	5	339
# contigs (≥0 bp)	5	5	339
# contigs (≥1,000 bp)	5	5	280
Largest contig	7,019,794	6,057,706	618,193
Total length	17,634,693	19,563,047	17,715,251
Total length (≥0 bp)	17,634,693	19,563,047	17,715,251
Total length (≥1,000 bp)	17,634,693	19,563,047	17,672,538
N50	3,774,987	4,538,514	156,673
N90	3,391,888	4,449,981	41,894
auN	4,909,996	4,963,904	190,940
L50	2	2	34
L90	4	4	116
GC (%)	34.02	34.58	33.79
# *N*'s per 100 kbp	0.06	0	1,284.75
# *N*'s	10	0	227,597

The new assembly is slightly shorter than the reference but significantly superior in terms of contig count and N50. The GC content, considered species-specific, is closer to that of the reference sequence than to the *M. capitatus* strain.

In 2014, Vaux et al. released the first draft genome CNRMA 12.647, consisting of 339 contigs, obtained by Illumina WGS of a panel of 18 samples—17 French clinical isolates from blood, sputum, and fecal samples collected between 2006 and 2012, along with the American strain CBS425.71. The genome was described as highly monomorphic (Vaux et al. 2014; Noster et al. 2022). The original Illumina-based FASTQ files of 18 sequences from Vaux et al. (2014) were downloaded from the European Nucleotide Archive (ENA) (Leinonen et al. 2011), together with 27 Illumina-based FASTQ files from the 2020 study by Menu et al. (2020). A sequence-based consensus was obtained using the VRMC001 sequence as a reference. After mapping, 8 samples from Vaux et al. (2014) were excluded from the phylogenetic analysis, as they contained only single-end sequences and had more than 3 million *N*s in the final assembly. The Illumina-based FASTQ files of the other 2 sequenced strains, VRMC002 and VRMC003, were also mapped onto the new sequence.

Vaux et al. (2014) established a basic single-nucleotide polymorphism (SNP) typing scheme, which led to the identification of 2 clades (A and B). However, further analysis by Noster et al. (2022) on *M. clavatus* isolates found no strains belonging to either clade A or clade B. Furthermore, Menu et al. (2020) were able to replicate the results of Vaux et al. (2014) but did not find any of their own sequences belonging to either clade A or B, even though they identified a third clade, C.

**Fig. 3.**
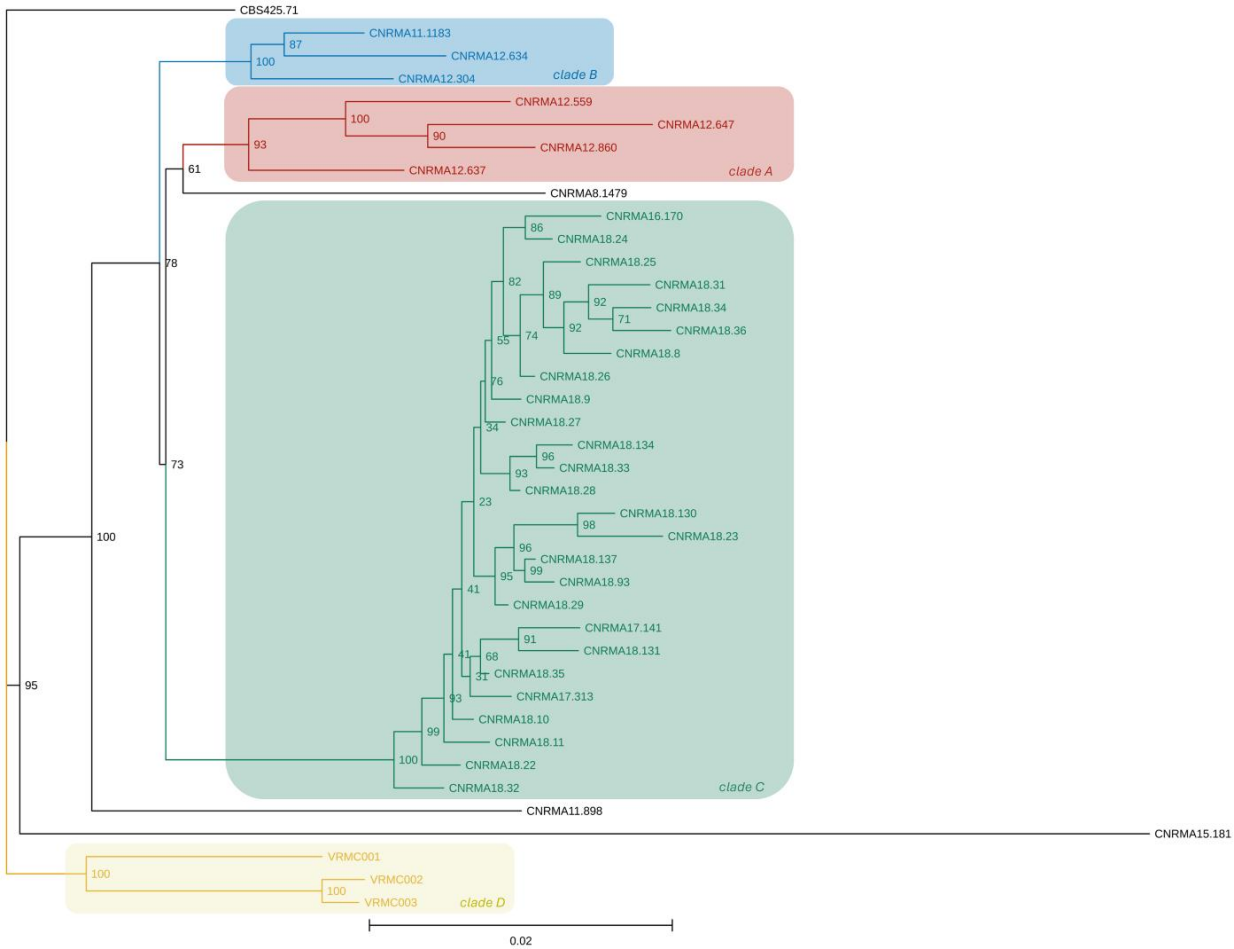
Maximum likelihood phylogenetic tree with bootstrap values obtained with IQ-TREE, based on 946 filtered SNPs from 40 sequences: 10 sequences from the French outbreak described by Vaux et al. (2014), 27 from the outbreak reported by Menu et al. (2020), and 3 sequences from this study. Each colored box represents a different clade: clade A in red, clade B in blue, clade C in green, and the newly proposed clade D in yellow. Strains shown in black are unassigned. The CBS 425.71 strain is the type specimen (USA 1971).

In this study, a SNP-based phylogenetic analysis was also performed. Only SNPs obtained from regions with a minimum of 20 reads mapped in each orientation, a mapping quality of at least 50, and supported by at least 80% of the reads were included in the analysis. Phylogenetic analysis conducted on 40 samples did not allow the assignment of any of our isolates to the previously described clades. Therefore, we propose a fourth clade D, including our 3 isolates VRMC001, VRMC002, and VRMC003. The maximum likelihood tree of the phylogenetic analysis is shown in Fig. 3.

In the next series of experiments, susceptibility tests were performed on all strains for specific drugs including anidulafungin, micafungin, caspofungin, flucytosine, posaconazole, voriconazole, fluconazole, and amphotericin. They all turned out to be resistant to both echinocandins and fluconazole. Minimum inhibitory concentrations (MICs) are reported in Table 3.

**Table 3.** Isolates information and MICs.

Isolate ID Isolation date Source	VRMC001 10/06/2024 Blood		VRMC002 01/07/2024 Bronchial aspirate		VRMC003 24/10/2024 Bronchial aspirate	
	MIC value	Interpretation	MIC value	Interpretation	MIC value	Interpretation
Anidulafungin	2	R^a^	2	R^a^	2	R^a^
Micafungin	2	R^a^	2	R^a^	2	R^a^
Caspofungin	>8	R^a^	>8	R^a^	>8	R^a^
Flucytosin	0.5	…^b^	0.25	…^b^	0.25	…^b^
Posaconazole	1	…^b^	1	…^b^	0.5	…^b^
Voriconaziole	1	…^b^	1	…^b^	0.5	…^b^
Fluconazole	64	R^a^	64	R^a^	64	R^a^
Amphotericin	2	…^b^	1	…^b^	2	…^b^

^a^Resistant.

^b^No breakpoint available.

**Fig. 4.**
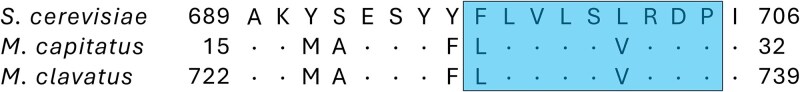
Protein-coding sequence alignment of the FKS HS1 gene of *S. cervisiae* (AY395693.1), *M.* c*apitatus* (KY783948.1), and *M. clavatus*. The blue box represents the alignment of the FKS HS1 region—dots indicate identity with the *S. cerevisiae* sequence, while letters denote substituted amino acids. The same F-to-L mutation identified in *M. capitatus* by Arrieta-Aguirre et al. (2018) that conferred echinocandin resistance was found in position 730 of the *M. clavatus* 1,3-β-glucan synthase.

Echinocandin resistance is driven by numerous substitutions affecting the drug target, the gene FKS Hot Spot 1 (HS1), which encodes for a catalytic subunit of the 1,3-β-glucan synthase complex (Lee et al. 2023). According to our annotation, the coding sequence for the 1,3-β-glucan synthase is in chromosome 1, in positions 4,010,420 to 4,016,359. Our sequences were examined for mutations at this specific site, revealing the same substitution previously observed in *M. capitatus* strains with reduced echinocandin susceptibility (Arrieta-Aguirre et al. 2018). The 3 strains present the same phenylalanine substitution to leucine and serine substitution to valine observed in the aforementioned study, on residues 20 and 24 of the encoded protein, respectively. Protein sequence mutations are represented in Fig. 4.

As with echinocandin resistance, azole resistance involves alterations in genes encoding the drug target 14-alpha-demethylase—*ERG11* in yeasts and *cyp51* in molds. In *C. albicans*, more than 140 amino acid substitution sites have been identified on *ERG11* (Lee et al. 2023).

Taking this into account, we searched the *M. clavatus* genome for putative homologs of *ERG11* to identify potential mutations or genetic alterations that could explain the observed resistance phenotype. In the VRMC001 genome, an automatically annotated hypothetical protein (identified using Funannotate and located between positions 1,383,540 and 1,385,204 on chromosome 1) exhibited 98% BLAST query coverage and 74.91% sequence identity with the 14-alpha sterol demethylase of *Geotrichum candidum* (NCBI accession number WZW60862.1). SWISS-MODEL predictions also identified the 14-alpha-demethylase of *Magnusiomyces paraingens* (template A0A5E8BCZ7.1.A) as the best template for modeling, having a 98% sequence coverage and 78% sequence identity to the query. The same sequence was identified in VRMC002 and VRMC003. We searched for active site mutations known to decrease azole susceptibility in *C. albicans* (Y140F/H) (Sagatova et al. 2016). Intriguingly, the active site of the *cyp51* homologue in *M. clavatus* retains the same amino acid residues found in wild-type *C. albicans ERG11*, questioning its involvement in azole resistance in *Magunsiomyces*. Further studies are needed to determine whether other changes in this or other proteins could impair fluconazole activity and contribute to azole resistance in *M. clavatus* (Sanglard et al. 1998; Marichal et al. 1999).

**Fig. 5.**
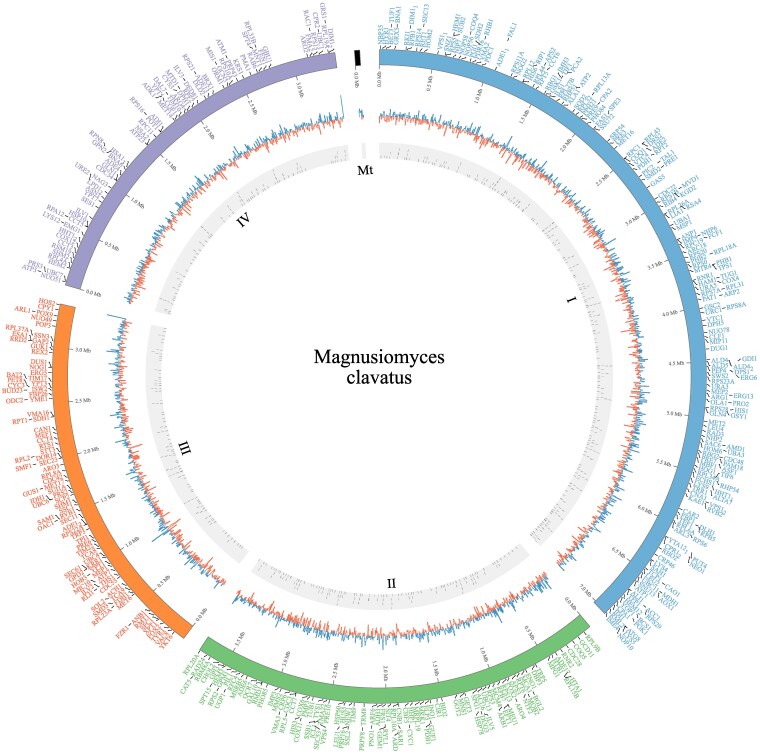
Circos plot of *M. clavatus* genome. In the external circle: gene names mapped with different colors on each chromosome (chromosome 1 in black, chromosome 2 in green, chromosome 3 in red, and chromosome 4 in purple); in the middle circle: GC content; in the inner circle: gene localization.

**Fig. 6.**
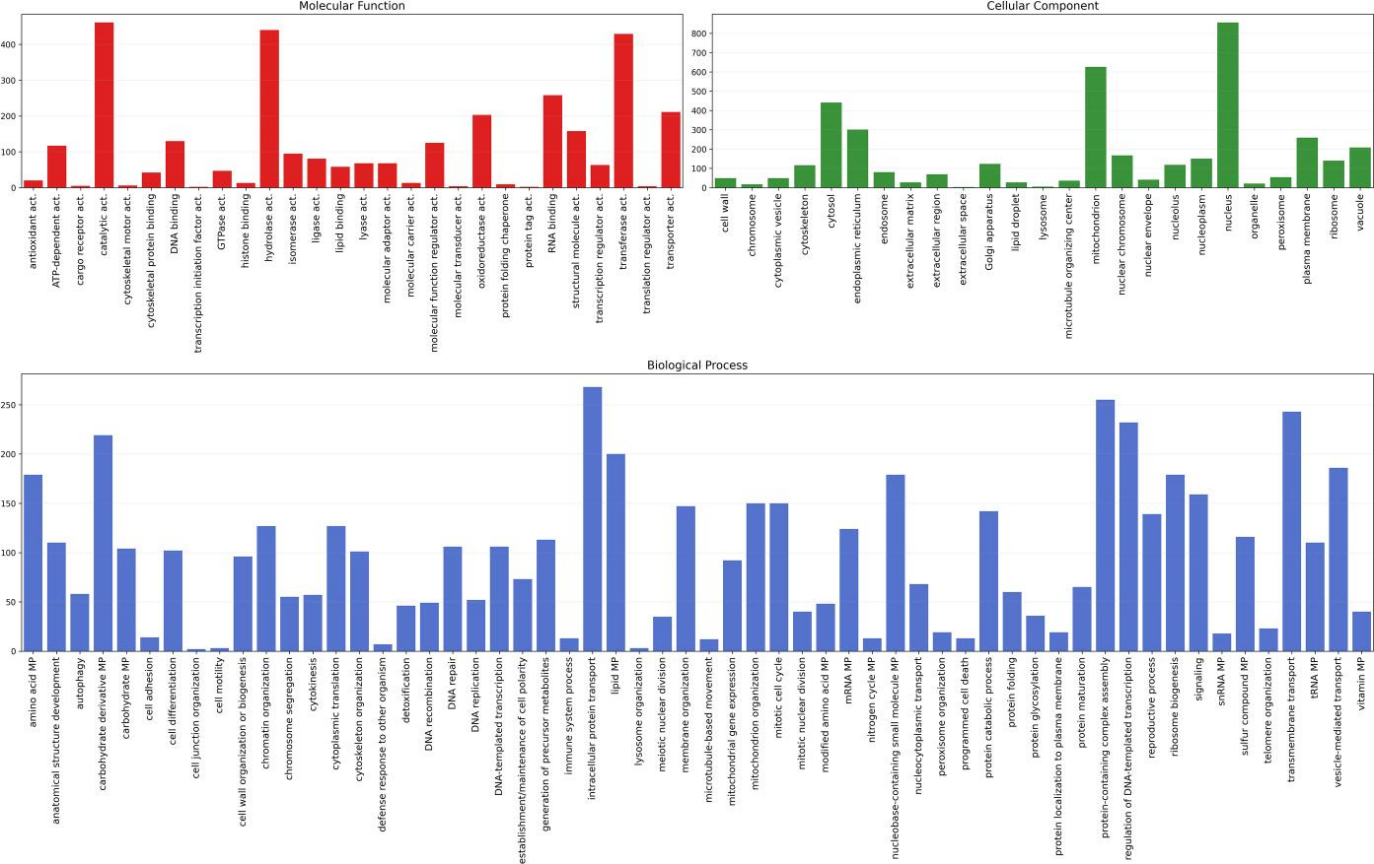
GO terms counts divided into the 3 GO aspects: molecular function, cellular component, and biological process. act., activity; MP, metabolic process.

**Fig. 7.**
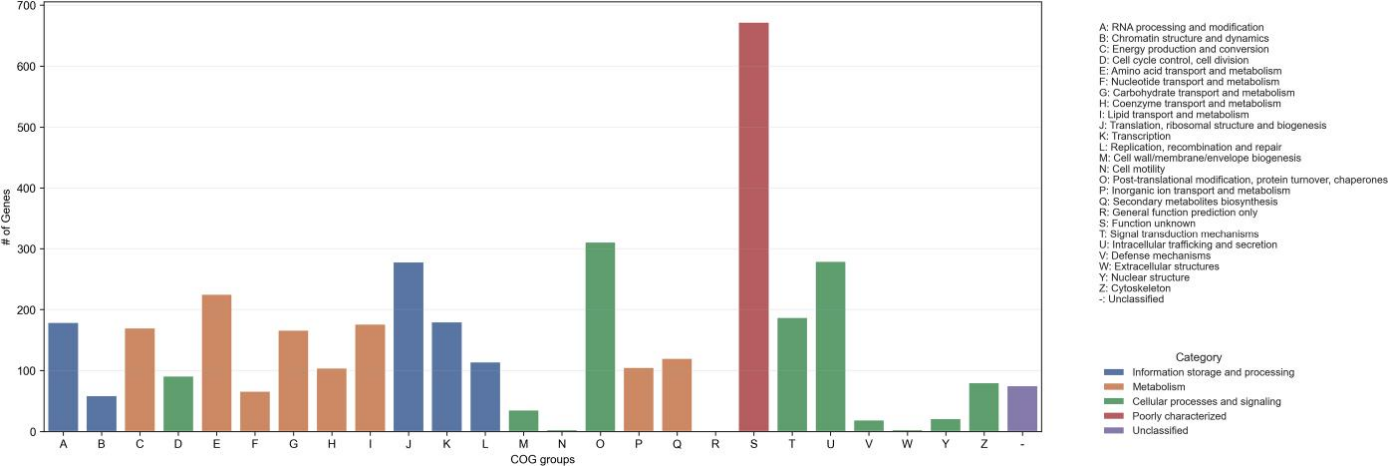
COG feature annotation classification chart for the *M. clavatus* genome. Each COG group was classified by macro-categories, represented by different colors: information storage and processing (blue), metabolism (orange), cellular processes and signaling (green), and poorly characterized (red). Unclassified genes are reported in purple.

Gene prediction performed on VRMC001 identified 4,171 gene models, including 4,061 protein-coding genes and 106 tRNAs. Exon analysis revealed 7,266 exons. A total of 1.984 transcripts were multi-exonic, while 2,081 were single-exon transcripts. Among the predicted coding sequences, 4,049 were considered complete, with only 7 missing start codons and 9 missing stop codons. A total of 3,090 gene annotations were pulled from the Pfam protein database, 84 genes from the carbohydrate-active enzyme (CAZyme), and 153 genes from the MEROPS protease database. Only 559 were assigned to already existing genes, represented in Fig. 5 in a Circos plot. A total of 12,632 Gene Ontology (GO) terms were identified and classified under one of the 3 GO aspects: molecular function, cellular component, and biological process (Fig. 6). All protein-coding genes were also classified according to the COG feature annotation and displayed in Fig. 7.

## Discussion

*M. clavatus* is an opportunistic fungal pathogen that poses a serious threat to immunocompromised patients. Despite the high mortality rate (reaching up to 80% in some patient groups), many biological aspects of this microorganism remain poorly understood. For a successful treatment outcome, early diagnosis and addressing the underlying immunosuppressive condition are of paramount importance (Buchta et al. 2019). Therefore, knowledge of the complete genome of this emerging pathogen may constitute the basis for the development of new molecular diagnostic tests.

As already mentioned, a chromosomal-level genome of *M. capitatus* has been available since 2018 (Brejová et al. 2019). However, despite the growing clinical relevance of *M. clavatus*, a complete genome for this species has remained unavailable. This study provides the resolved *M. clavatus* genome assembly. We described the *M. clavatus* genome organized into 4 nuclear chromosomes and a mitochondrial genome. This organization mirrors that of *M. capitatus* (Brejová et al. 2019), although notable structural differences are detectable between the 2 species. The genome of *M. clavatus* is smaller in size (17.6 Mb) compared to the approximately 19.6 Mb genome reported for *M. capitatus*.

Further phylogenetic analysis was performed on the 3 strains by analyzing sequencing data obtained from previous studies. Vaux et al. (2014) initially described 2 main clades (A and B) of *M. clavatus*, while later studies (Menu et al. 2020) proposed the existence of a third clade (C). Our phylogenetic analysis did not place our isolates within any of these known clades. Therefore, a new clade (D) for the 3 samples described in this study is proposed.

The antifungal susceptibility profile of our isolates confirmed intrinsic resistance to echinocandins, aligning with previous findings on *M. clavatus* and *M. capitatus*. Notably, a key amino acid substitution was identified in the region corresponding to the 1,3-β-glucan synthase complex, resembling the mutation found in *M. capitatus* strains, which are intrinsically resistant to echinocandins. The same F-to-L mutation in *C. albicans* FKS at position 641 has been previously associated with echinocandin resistance (Garcia-Effron et al. 2009). In *M. clavatus*, the L730 in the 1,3-β-glucan synthase complex—located in the analog of FKS HS1—supports the hypothesis that alterations in these amino acids may contribute to antifungal resistance in *Magnusiomyces* species, presenting a major challenge for treatment. In contrast, the L-to-V substitution at residue 735 still has an unknown impact when observed in other fungal species. (Arrieta-Aguirre et al. 2018).

On the other hand, the exact mechanism of azole resistance in *M. clavatus* remains unclear. A homologue of the *cyp51* gene—encoding for a 14-alpha sterol demethylase, the target of antifungal azoles—was identified in this species. In other fungi, resistance has been associated with modification of this enzyme (Sanglard et al. 1998; Marichal et al. 1999). However, whether fluconazole resistance in *M. clavatus* is similarly driven by alterations in this protein has yet to be demonstrated.

*M. clavatus* exhibits additional features that differentiate it from other fungi, including its ability to colonize the gastrointestinal tract asymptomatically, its environmental persistence in nosocomial settings, and its potential epidemiological link to specific outbreaks (Vaux et al. 2014; Menu et al. 2020). This study provides the chromosomal-level genome assembly of *M. clavatus*, giving a deeper insight into its genetic structure, phylogenetic diversity, and antifungal resistance mechanisms. This information lays the groundwork for future investigations into the biology, pathogenicity, and epidemiology of this emerging opportunistic pathogen and could lead to improved diagnostic and new therapeutic strategies.

## Supplementary Material

jkaf201_Supplementary_Data

## Data Availability

The fasta sequences that support the findings of this study, VRMC001, VRMC002, and VRMC003, are uploaded using the GSA Figshare portal (https://doi.org/10.25387/g3.29350325) and are available in NCBI at https://www.ncbi.nlm.nih.gov, with accession numbers GCA_051013725.1, GCA_051013875.1, and GCA_051013885.1, respectively. Script used to build the vcf files is available at https://github.com/et-univr/VCFtoAlignfasta2. The raw data of this article are available in NCBI under BioProject accession code PRJNA1220052, at BioSample SAMN46708367, SAMN48134874, and SAMN48134957. Supplemental material available at *G3* online.
