# Supplementary material for: Chromosomal-level assembly of Magnusiomyces clavatus: novel genetic insights on an emerging fungal pathogen
Source: G3 (Bethesda). 2025 Sep 3;15(11):jkaf201. doi: 10.1093/g3journal/jkaf201 (PMC12611253; doi:10.1093/g3journal/jkaf201)
Supplement: jkaf201_Supplementary_Data [file jkaf201_supplementary_data.zip › Supplementary_Figures_Legend_G3-2025-405997.docx]

**Supplementary Figures Legend**

**Supplementary Figure 1** Raw MIC data of the Sensititre™ YeastOne YO10 kit susceptibility testing for VRMC001 (panel a), VRMC002 (panel b) and VRMC003 (panel c).

**Supplementary Figure 2** GenomeScope visualization of the *M. clavatus* Illumina FASTQ data. Raw genome size is estimated to be 18.9 Mb prior to the assembly steps.

**Supplementary Figure 3** Merqury k-mer count (k-mer of 21 nucleotides) of the Illumina reads and the final assembly. The small bar in the left corner corresponds to k-mers present only in the assembly and then interpreted as artifacts.

**Supplementary Figure 4** Bandage representation of the mitochondrial genome. The regions shared with the mitochondrial genome of *M. capitatus* according to BLAST are highlighted in blue.
