# Supplementary figures and images for: Chromosomal-level assembly of Magnusiomyces clavatus: novel genetic insights on an emerging fungal pathogen
Source: G3 (Bethesda). 2025 Sep 3;15(11):jkaf201. doi: 10.1093/g3journal/jkaf201 (PMC12611253; doi:10.1093/g3journal/jkaf201)

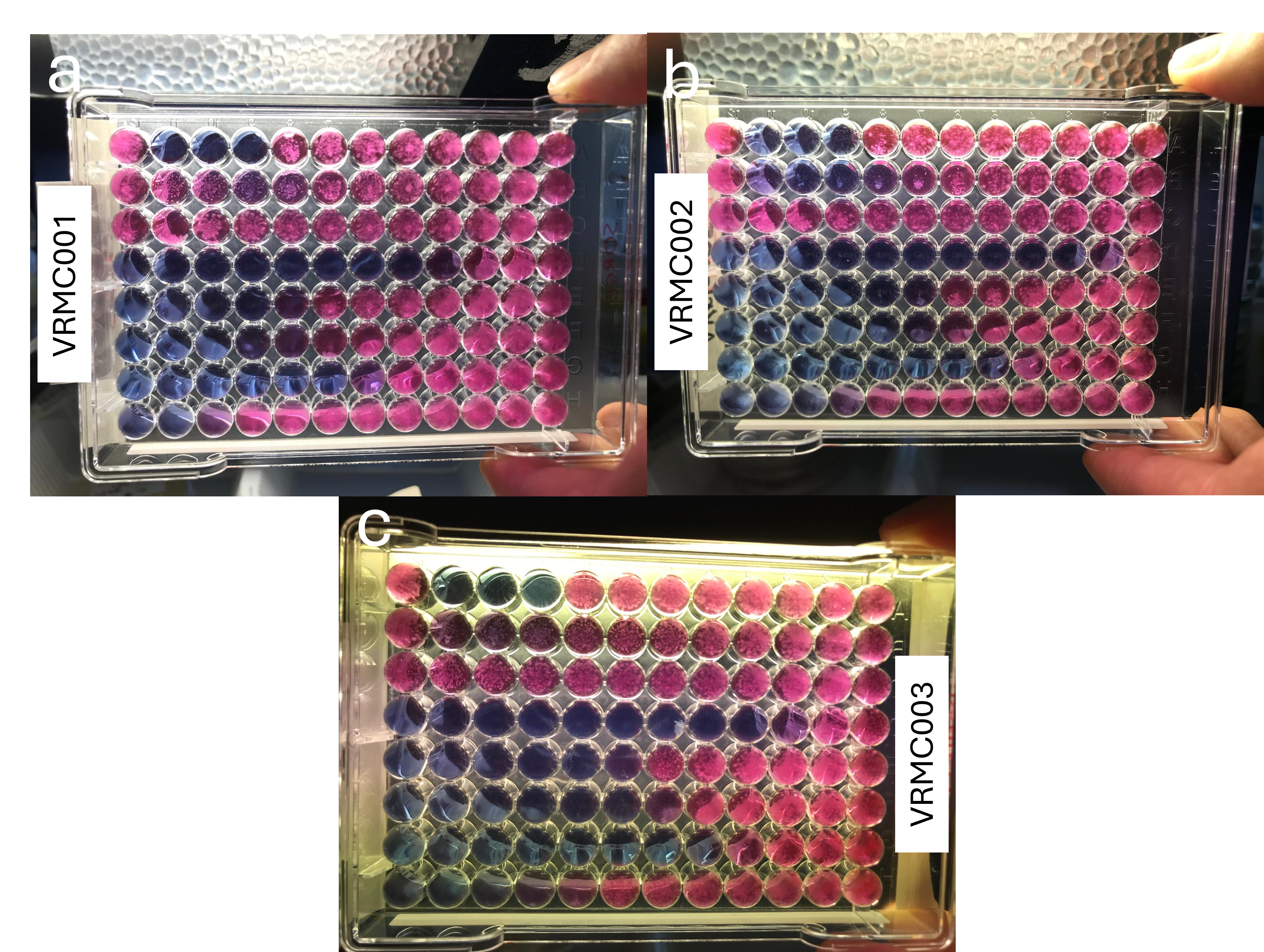

Supplement: jkaf201_Supplementary_Data [file jkaf201_supplementary_data.zip › Supplementary_Figure_1_G3-2025-405997.png]

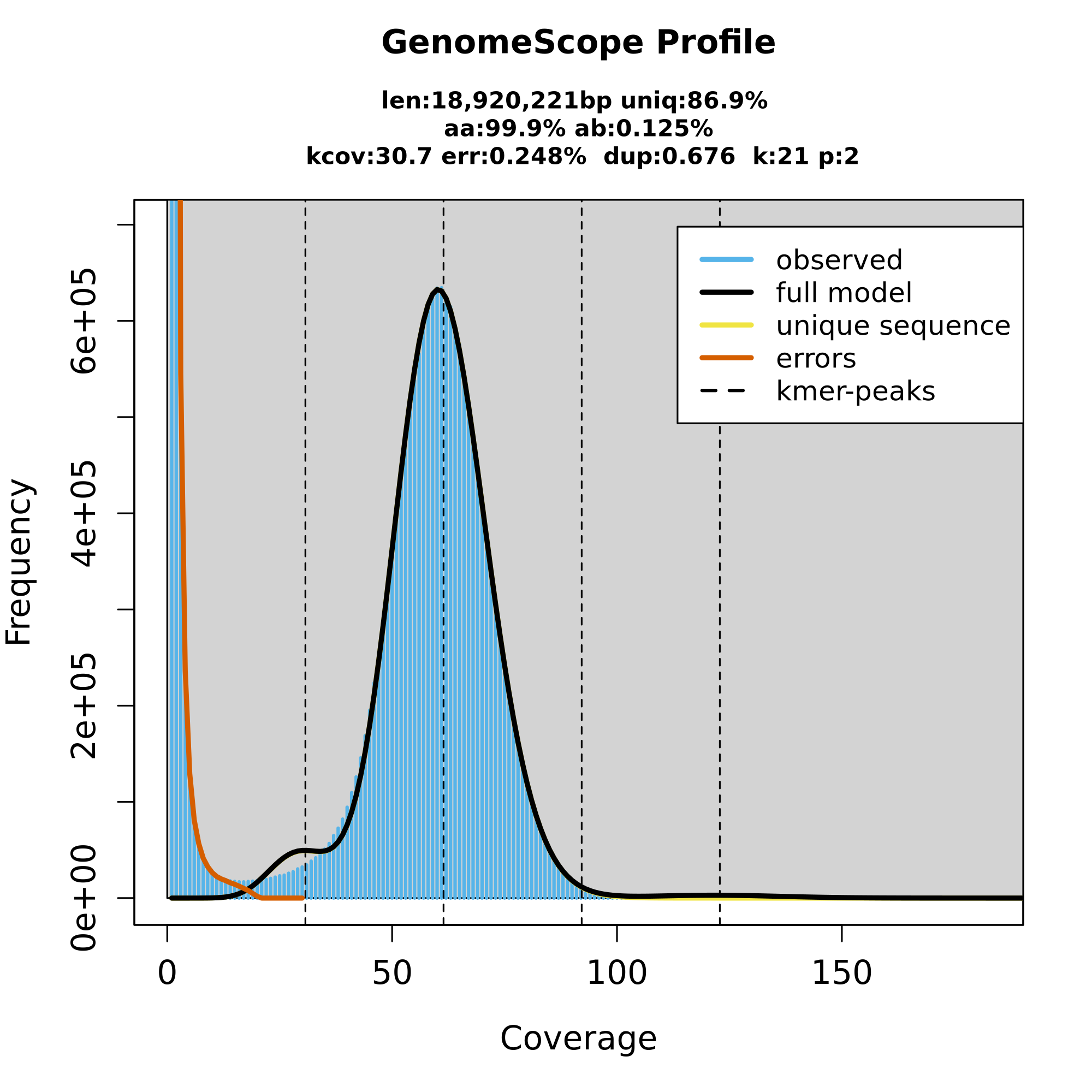

Supplement: jkaf201_Supplementary_Data [file jkaf201_supplementary_data.zip › Supplementary_Figure_2_G3-2025-405997.png]

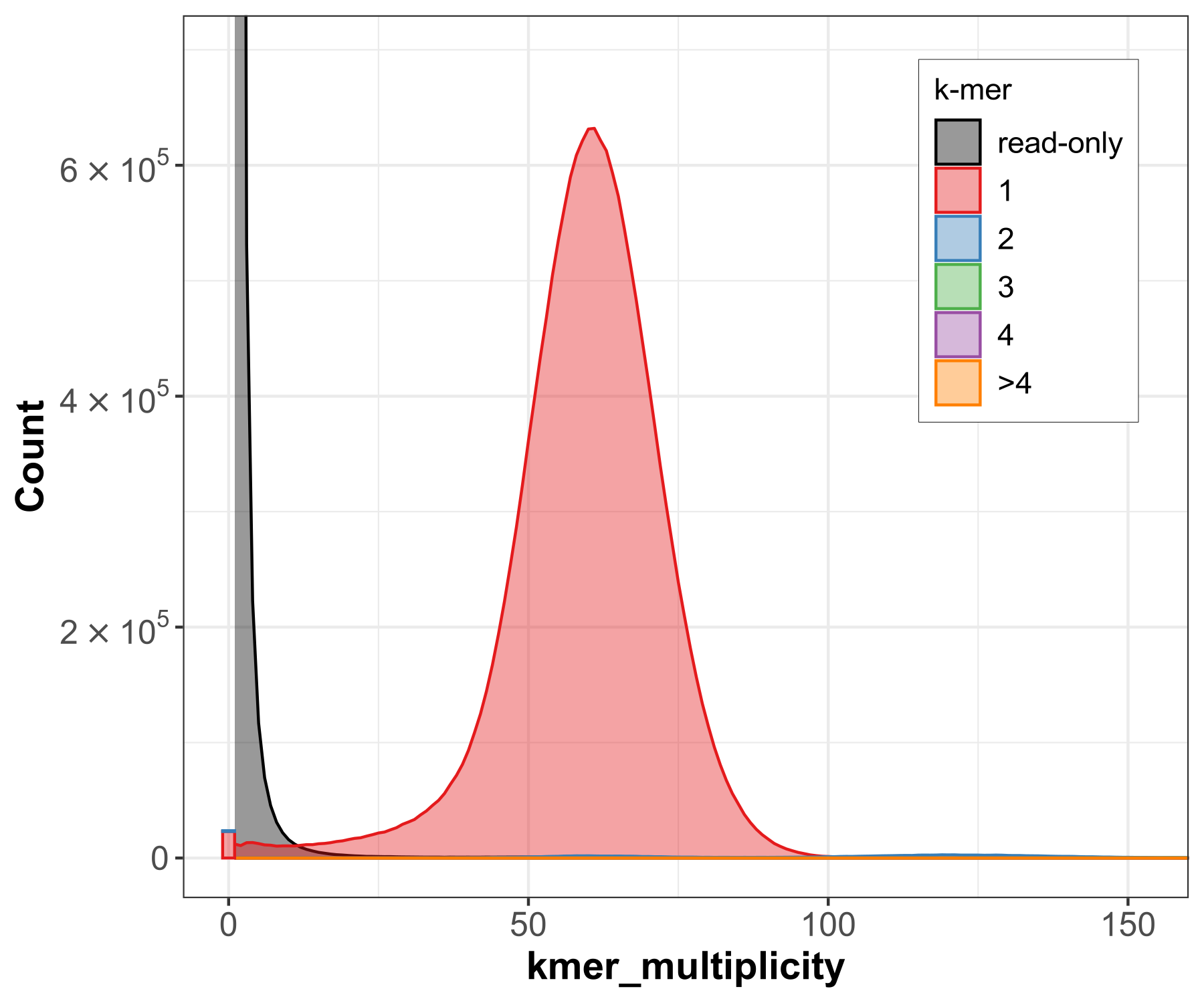

Supplement: jkaf201_Supplementary_Data [file jkaf201_supplementary_data.zip › Supplementary_Figure_3_G3-2025-405997.png]

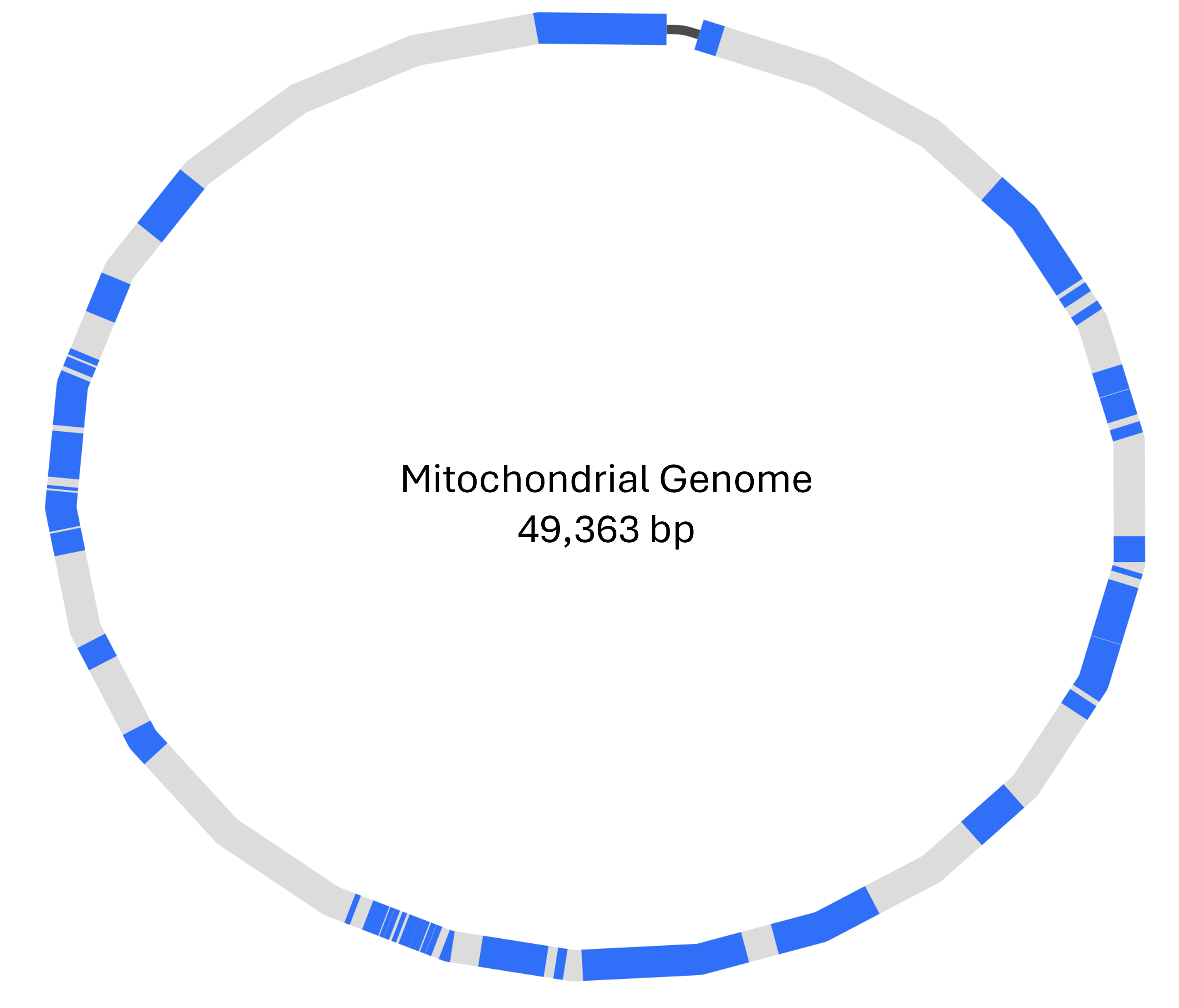

Supplement: jkaf201_Supplementary_Data [file jkaf201_supplementary_data.zip › Supplementary_Figure_4_G3-2025-405997.png]
